# Proton Adsorption Selectivity of Zeolites in Aqueous Media: Effect of Si/Al Ratio of Zeolites

**DOI:** 10.3390/molecules191220468

**Published:** 2014-12-08

**Authors:** Moses Wazingwa Munthali, Mohammed Abdalla Elsheikh, Erni Johan, Naoto Matsue

**Affiliations:** 1 Department of Life Environmental Conservation, Faculty of Agriculture, Ehime University, 3-5-7 Tarumi, Matsuyama 790-8566, Japan; E-Mails: Johan@agr.ehime-u.ac.jp (E.J.); matsue@agr.ehime-u.ac.jp (N.M.); 2 Department of Soil and Environment Sciences, Faculty of Agriculture, University of Khartoum, Shambat, P.O. Box 32, Khartoum North 13314, Sudan; E-Mail: mohmedelsheikh@gmail.com

**Keywords:** zeolite, Si/Al ratio, negative charge density, H^+^ selectivity, cation adsorption

## Abstract

In addition to their well-known uses as catalysts, zeolites are utilized to adsorb and remove various cations from aqueous system. The adsorption of the cations is ascribed to the negative charge of zeolites derived from isomorphous substitution of Si by Al. The amount of Na^+^ adsorption on 4A, X, Y, Na-P1 and mordenite type zeolites were determined in aqueous media, in a two-cation (Na^+^ and H^+^) system. Although each zeolite has a constant amount of negative charge, the amount of Na^+^ adsorption of each zeolite decreased drastically at low pH−pNa values, where pH−pNa is equal to log{(Na^+^)/(H^+^)}. By using the plot of the amount of Na^+^ adsorption *versus* pH−pNa, an index of the H^+^ selectivity, which is similar to the pKa of acids, of each zeolite was estimated, and the index tended to increase with decreasing Si/Al ratio of zeolites. These indicate that zeolites with lower Si/Al and higher negative charge density have higher H^+^ adsorption selectivity, and in fact, such a zeolite species (4A and X) adsorbed considerable amount of H^+^ even at weakly alkaline pH region. The adsorption of H^+^ results in the decrease of cation adsorption ability, and may lead to the dissolution of zeolites in aqueous media.

## 1. Introduction

Zeolites, a major group of inorganic cation exchange materials, are of great interest as adsorbents and catalysts due to their high negative charge density and high selectivity toward some kinds of cations [1,2]. In aqueous media, they are widely used in a wide range of pH values for industrial applications such as detergency and water treatment, removal of radionuclides from nuclear waste effluents [3], treatment of mine acid water drainage, decontamination or remediation of heavy metals [4], as fertilizer and pesticide carriers in nano-organic composites, amelioration of acidic soils and in the oil refining and petrochemical industry [5]. Usually, the cation retention ability of zeolites is evaluated based on their cation exchange capacity (CEC). The CEC of zeolites has been assumed to be equivalent to the amount of negative charge resulting from the isomorphous substitution of Si by Al, and regarded to be constant at different pH with ascribing or inferring to the negative charge of 2:1 layer silicates clay minerals such as montmorillonite [6]. 

Recently, we reported that the CEC (the amount of Na^+^ retention) of Na^+^-saturated 4A and Na-P1 type zeolites decreased with the decrease in equilibrium pH even in the pH 7 to 9 region. The decrease in the CEC with the decrease in equilibrium pH was due to strong adsorption selectivity of H^+^ toward the negative charges of zeolites [7]. The decrease in the retention amount of Na^+^ due to the adsorption of H^+^ also implies the reduction in the retention ability of the zeolites cations other than H^+^. Currently, which physicochemical factors of zeolites are significantly influencing H^+^ adsorption selectivity in relation to the decrease of CEC at different pH it is not yet well understood. It is likely that the degree of H^+^ adsorption selectivity among various kinds of zeolite species at different pH varies significantly depending on the prevailing physicochemical factors of their crystal structural frameworks [8].

Various zeolites have different crystal structures arising from variations in composition, distribution and ordering of S–O–Al or Si–O–Si linkages in their structural framework [9]. Differences also exist in the Si/Al ratio within and among various kinds of zeolite species which result in variations in the location, amount and distribution of negative charge density in the structural frameworks, cages or pores of different diameters, nature or absence of hydration water or other ligands and presence and position of extraframework cation(s) [3,10,11,12,13]. Usually such factors affect the cation retention behavior and acid strength of the Si(OH)Al group in various zeolites [14]. It has been shown that the adsorption or selectivity of some cations such as Na^+^, Ni^2+^ varies significantly among different zeolites [15]. 

Many studies have been conducted on ionic selectivity in zeolites in relation to catalytic property [16], hydrogen storage [17,18] and dissolution of zeolites [19,20]. However, there is scarce information on H^+^ adsorption selectivity or decrease in CEC of various kinds of zeolites. As such, factors affecting H^+^ adsorption selectivity of various zeolites at different pH are not yet well known. Recently, we have been evaluating a number of factors that influence H^+^ adsorption selectivity in relation to CEC decrease in various zeolites at different pH values. In this paper, we present the effect of charge density, Si/Al ratio, types of crystal structure on H^+^ adsorption selectivity and CEC decrease of Na^+^-saturated zeolites at different pH−pNa, where pH−pNa is a common logarithm of the ratio of activities of Na^+^ to H^+^, log{(Na^+^)/(H^+^)}. The zeolite samples used were commercially available 4A, X, Y, and mordenite type zeolites, and four synthetic Na-P1 type zeolites with different Si/Al ratio.

## 2. Results and Discussion

### 2.1. Stability of Zeolites with CEC Measurement

The dissolution of zeolites in aqueous media at high temperature [19] and under strong acidic conditions [20] has been reported. Although the present CEC measurement was conducted at room temperature and in a moderately acidic to moderately alkaline pH region, the dissolution of the zeolite samples should be checked. In the present CEC measurements, each zeolite sample was put into HCl solutions with various concentrations, distilled water, or NaOH solutions with various concentrations. The pH values after 3 h of shaking, namely equilibrium pH, are shown in Table 1 for all cases examined: the lowest pH was 2.56 for mordenite type zeolite, and the highest pH was 11.37 for Y type zeolite. In Table 1, pH values when zeolite samples were put into distilled water were indicated by bold characters.

**Table 1:** pH and Na^+^ concentration at equilibrium, pH−pNa and CEC of zeolites.

Zeolite	Si/Al	pH ^1^	[Na^+^] ^2^	pH−pNa ^3^	CEC ^4^
4A	1.00	11.36	0.26	9.05	565
		10.85	0.47	8.04	560
		10.16	0.68	6.99	558
		**10.05**	**0.69**	**6.88**	**556**
		9.26	0.92	6.23	553
		8.49	1.94	5.78	532
		7.87	2.55	5.28	520
		7.23	4.98	4.90	471
		6.91	6.04	4.68	450
		6.64	7.65	4.55	418
X	1.24	11.29	0.96	8.99	461
		10.65	1.17	7.99	457
		**9.84**	**0.63**	**7.22**	**440**
		9.64	1.53	6.81	449
		9.40	1.79	6.63	444
		8.28	3.19	5.75	416
		7.89	4.47	5.51	391
		7.49	5.86	5.22	363
		7.10	7.86	4.95	323
		6.97	9.30	4.89	294
		6.79	10.92	4.78	262
Y	2.66	11.37	0.02	9.03	250
		11.09	0.13	8.45	247
		10.37	0.40	6.96	250
		9.18	0.03	5.64	245
		8.38	0.04	4.87	243
		**7.41**	**0.05**	**3.89**	**240**
		7.01	0.19	3.29	236
		6.03	0.79	2.91	224
		5.61	2.21	2.93	196
		5.34	3.20	2.82	176
		5.15	3.93	2.71	162
		5.01	5.57	2.72	129
		4.81	7.94	2.67	81
Na-P1	1.10	**10.21**	**0.28**	**6.40**	**478**
		8.54	0.48	4.90	476
		7.61	1.15	4.24	470
		6.88	3.18	4.13	459
		6.60	4.18	3.84	449
		6.19	4.54	3.63	409
		6.03	6.67	3.54	367
Na-P1	1.67	11.36	0.35	9.01	424
		10.75	0.28	7.88	424
		**10.08**	**0.15**	**5.95**	**429**
		9.11	0.21	5.42	426
		8.34	0.71	5.17	416
		7.48	0.72	4.32	416
		6.46	1.78	3.67	394
		5.93	2.92	3.36	372
		5.54	3.79	3.09	354
		5.22	5.01	2.89	330
		4.98	6.60	2.76	298
Na-P1	1.80	**10.36**	**0.14**	**6.51**	**397**
		7.98	0.28	4.41	394
		6.86	0.61	3.63	387
		6.26	1.11	3.29	377
		5.76	1.84	3.00	363
		5.43	3.12	2.90	337
		5.17	4.24	2.77	315
Na-P1	2.30	**9.96**	**0.23**	**6.30**	**314**
		6.81	0.40	3.40	310
		5.66	0.56	2.69	307
		5.08	1.93	2.34	279
		4.72	3.12	2.19	256
		4.44	4.92	2.10	220
		4.33	6.25	2.09	193
morde-nite	4.88	11.31	0.35	9.00	179
		10.72	0.31	7.86	179
		**7.97**	**0.78**	**4.85**	**176**
		5.84	0.15	2.01	176
		4.86	0.39	1.44	173
		4.38	0.54	1.10	171
		4.11	0.72	0.96	168
		3.75	1.42	0.89	153
		3.28	2.51	0.66	131
		3.01	3.55	0.53	110
		2.83	4.37	0.43	94
		2.67	4.76	0.31	86
		2.56	5.09	0.23	79

^1^ equilibrium pH; ^2^ equilibrium Na^+^ concentration (mM); ^3^ pNa: negative logarithm of Na^+^ activity; ^4^ cation exchange capacity or amount of Na^+^ retention (cmol·kg^−1^; cmol is centimol).

**Figure 1:**
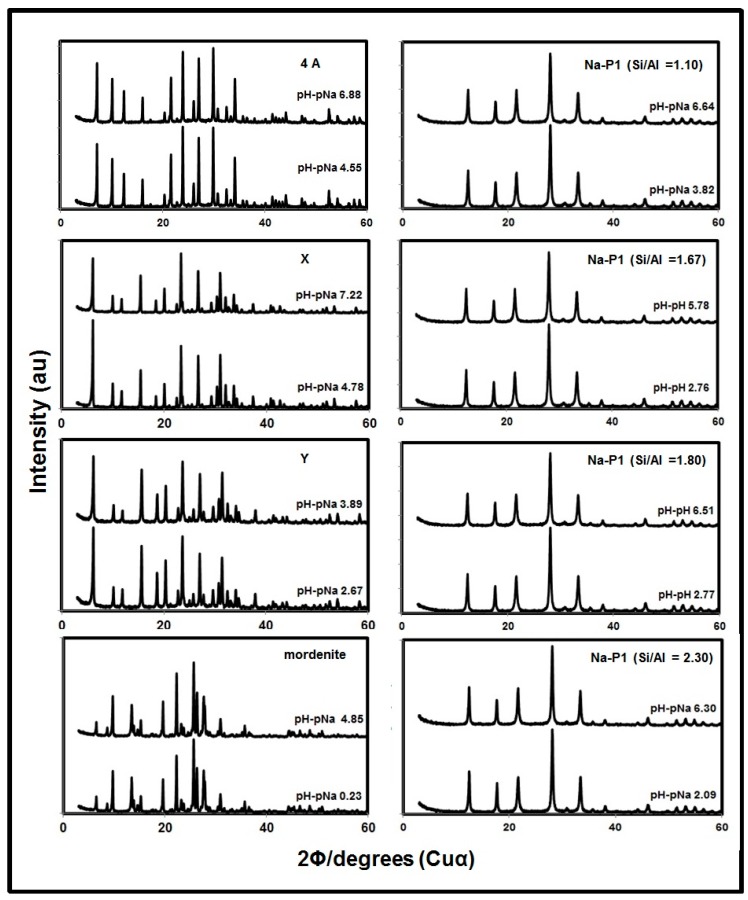
XRD patterns of zeolites after CEC measurement at different pH–pNa.

**Figure 2:**
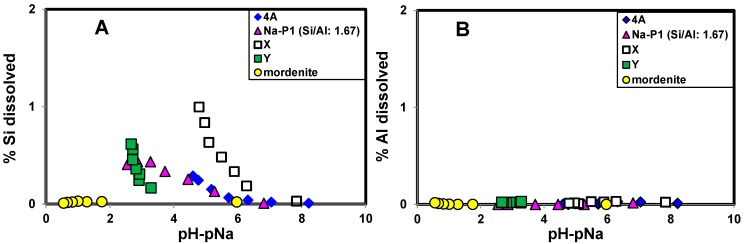
Effect of pH−pNa on the dissolution of Al and Si from zeolites. (A) Plots of % Si dissolved. (B) Plots of % Al dissolved.

Figure 1 shows XRD patterns of each zeolite after the measurement of CEC with distilled water (lower) and with the highest HCl concentration (upper), and the analysis of the XRD patterns revealed that there was no change, no destruction and no formation of new materials after washing with the highest HCl concentration. The XRD patterns of each zeolite before the CEC measurement and after the measurement with NaOH were the same as that after the CEC measurement with distilled water (not shown). The amounts of dissolution of Al and Si with the CEC measurement were less than 0.5% for Al and 1.5% for Si, as shown in Figure 2. The dissolution of Al and Si was possibly due to the dissolution of amorphous aluminosilicate materials such as precursors of zeolites contained in the samples, because in some cases XRD peaks of zeolites became stronger after the CEC measurement. The dissolution of minuscule amounts of zeolites framework might also be occurred. In these cases, the dissolution of Al and Si occurred simultaneously, but the dissolved Al species, probably hydroxy-Al cations, might be readily absorbed on zeolites. This is the reason why the observed amount of Al dissolution was very low (Figure 2B). In conclusion, the amounts of dissolution of Al and Si from zeolites were very small compared to the changes in CEC (Table 1). However, in other preliminary experiments when we prolonged the shaking time for 7 days at around pH 6, 4A type zeolite was almost all dissolved and amorphous aluminosilicates were formed. This observation calls for a need of more deep studies on the dissolution of various zeolites under different pH conditions.

### 2.2. Effect of pH−pNa on Na^+^ Retention by Zeolites

When 1 g of a Na^+^-saturated zeolite was added into 200 mL of distilled water, a small amount of Na^+^ was released, and pH of the supernatant became alkaline, as shown in Table 1 with bold characters. This indicated that Na^+^ retained to compensate negative charge of zeolite was partly released to the aqueous phase, followed by the adsorption of H^+^ originated from the dissociation of water. This is a kind of hydrolysis, and the extent of Na^+^ release and H^+^ adsorption is related to the adsorption selectivity of zeolite to H^+^. When dilute HCl solution was added instead of distilled water, part of the added H^+^ replaced Na^+^ on the zeolite, and the rest remained in solution phase. As a result, with increasing concentration of HCl added, the solution pH decreased, Na^+^ concentration increased, and CEC or the amount of Na^+^ retention decreased in each zeolite (Table 1).

**Figure 3:**
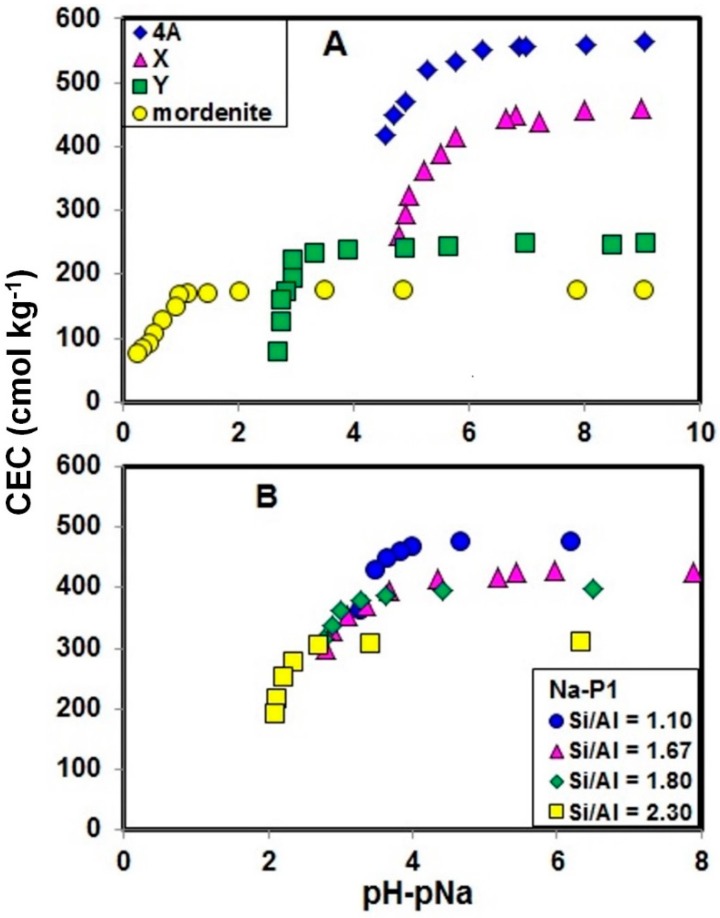
Change in CEC of zeolites with change in pH−pNa. (A) Plots of 4A, X, Y and mordenite type zeolites. (B) Plots of Na-P1 with different Si/Al ratio. The unit of CEC, cmol·kg^−1^, means centimole per kilogram.

Figure 3 shows change in CEC of 4A, X, Y, Na-P1 and mordenite type zeolites with changes in pH−pNa, and the plots were made up by using the data in Table 1. The values of CEC of all zeolites tended to decrease with a decrease in pH−pNa. At higher pH−pNa region, CEC of the zeolites were slightly affected by pH−pNa, and the CEC value of this region is equal to the amount of isomorphous substitution of Si by Al. As seen in Figure 3, CEC or the amount of Na^+^ retention of the zeolites were well expressed by pH−pNa or log{(Na^+^)/(H^+^)}, because in the present two-cations (Na^+^ and H^+^) system, the amount of Na^+^ adsorption for each zeolite is univocally determined by the ratio of the two cations [21].

With increasing concentration of HCl added, pH−pNa decreased, and CEC of the zeolites decreased with the decrease of pH−pNa. For zeolites with lower Si/Al ratio, 4A and X type zeolites, the CEC values began to decrease at higher pH−pNa, while for zeolites with higher Si/Al ratio, Y and mordenite type zeolites, the CEC curve remained constant or continued to plateau and began to decrease at very low pH−pNa. Considering the points of pH−pNa at which CEC began to decrease, they were significantly different among the zeolites. The decrease of CEC or the amount of Na^+^ retention of the five zeolite species indicates that the negative charge of the zeolites was partly compensated by the adsorption of H^+^. The adsorption of H^+^ onto zeolites is due to the high affinity of the zeolites toward H^+^, and Figure 3 reveals that the adsorption of H^+^ onto 4A and X type zeolites occurred even at higher pH−pNa values or at alkaline pH region (Table 1). It should also be noted that X and Y type zeolites have the same structural framework, namely faujasite type, but have different charge densities or Si/Al ratio, and the H^+^ adsorption selectivity of the two zeolites were different from each other.

The five zeolite species used have different structural frameworks, pore structures and Si/Al ratios. Here we examined the effect of Si/Al of zeolites on H^+^ adsorption selectivity using zeolite samples of the same structural frameworks but having different Si/Al ratio. For this purpose, X and Y type zeolites were compared first, because as mentioned above, the two zeolite species have the same faujasite-type structure but have different Si/Al ratios. Comparing the plot of CEC *versus* pH−pNa between X and Y type zeolites (Figure 3A), the CEC values of X type zeolite (Si/Al = 1.24) began to decrease from a pH−pNa of around 6, while for Y type zeolite (Si/Al = 2.66) the CEC curve was a plateau until a pH−pNa of around 4. When we look at pH value, the CEC of X type zeolite decreased from around pH 8, and the pH was around 6 for Y type zeolite. The observations indicate that faujasite type zeolites with lower Si/Al ratio have higher H^+^ selectivity. A similar trend was also observed for four synthetic Na-P1 type zeolite samples of the same structural framework but with different Si/Al ratio from 1.10 to 2.30 (Figure 3B). The CEC of Na-P1 type zeolite with lower Si/Al ratio began to decrease at higher pH−pNa while Na-P1 type zeolite with higher Si/Al began to decrease at lower pH−pNa. This indicated that Na-P1 type zeolite with lower Si/Al ratio have higher H^+^ adsorption selectivity.

Generally, acidity or H^+^ adsorption selectivity of a monovalent acid is expressed by a pKa value, which is equal to the pH value when 50% of its negative charge is deprotonated, and a greater pKa indicates weaker acidity and stronger H^+^ adsorption selectivity. When the degree of dissociation of a monovalent acid is plotted against pH, a sigmoid curve is obtained, and the curves in Figure 3 are similar to the acid dissociation curves, although the horizontal axis is pH−pNa. This is because the H^+^ adsorption selectivity of zeolites is affected by coexisting Na^+^, and this is different from the cases of low molecular mass acids in aqueous media. Then, to express the H^+^ selectivity of zeolites on a semi-quantitative level, we used the pH−pNa value where the CEC of each zeolite become 80% of the amount of isomorphous substitution of Si by Al, instead of pH−pNa at 50%, and the pH−pNa value is hereafter written as (pH−pNa)_80_: the amount of isomorphous substitution was assumed to be equal to the CEC obtained with the addition of distilled water (bold numbers in Table 1). Since CEC measurement until lower pH−pNa region was difficult due to the problem of dissolution of zeolites, except for Y and mordenite type zeolites, we adopted 80% instead of 50%.

**Figure 4:**
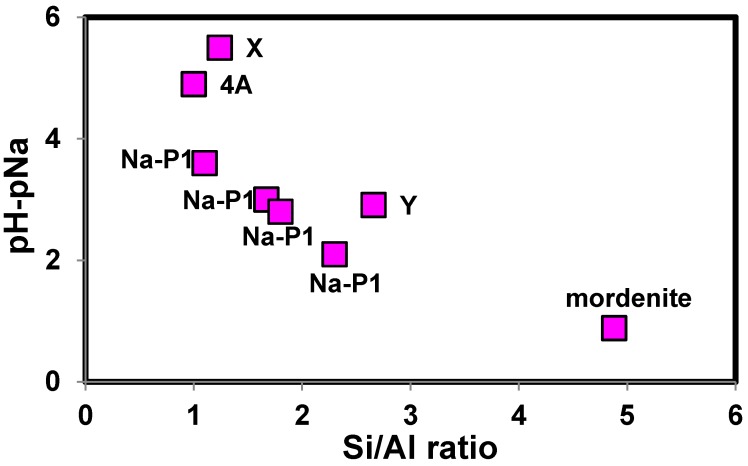
Correlation of (pH−pNa)_80_ with Si/Al ratio of zeolites.

Here, we assumed that the (pH−pNa)_80_ can be a measure of the H^+^ selectivity of a zeolite. When the (pH−pNa)_80_ values were plotted against Si/Al ratio of zeolites, a negative correlation was obtained (Figure 4), although four different types of zeolite frameworks were contained in the figure {Linde-type A (4A), faujasite type (X and Y), P type (Na-P1) and mordenite type}. Therefore Figure 4 indicated that irrespective of the difference in the zeolite framework types, Si/Al ratio is closely related to the H^+^ selectivity of a zeolite. However, data points of the four Na-P1 type zeolite samples made up a straight line, but it deviated from the others, and 4A type zeolite (Si/Al = 1.0) had lower (pH−pNa)_80_ value than X (Si/Al = 1.24) had. These indicated factors other than Si/Al ratio affected the H^+^ selectivity of the zeolites, and the factors are discussed in the following section.

### 2.3. Factors Affecting H^+^ Adsorption Selectivity

The Si/Al ratio was significantly different among the zeolite samples (4A: 1.00; X: 1.24; Y: 2.66; Na-P1: 1.10–2.30; mordenite: 4.88). Usually, changes in Al content lead to distortions of geometrical features such as Si–O–Al angle and bond length, and affect surrounding atoms such as structural framework atoms and extra-framework cation(s) [11]. Variations in Si/Al ratio generally result in differences in the amount and distribution of Si–O–Al groups in their crystal structures [12]. Consequently, as the Si/Al ratio changes within and among zeolite crystal structures, the behavior or properties of zeolites are significantly altered [22]. Zeolites with higher Si/Al ratio have more Si–O–Si linkages than Si–O–Al linkages, and *vice versa* for zeolites with lower Si/Al ratios. 

The Si–O–Al and Si–O–Si linkages are the ones partly playing a major role in influencing the degree of H^+^ adsorption selectivity among the zeolites as they determine bond angles and lengths around the acid site [8], the distance of nearby or surrounding Si–O–Al or Si–O–Si groups within or around the zeolite structural framework [23], and the types of guest molecules or surrounding atoms and electronic charge distribution in the structural framework [9]. These factors affect the electrostatic potential and the strength of binding interaction of cations on negative charge sites in the structural framework [24], and led to increased or decreased H^+^ selectivity. Zeolites possess a number of acidic and basic sites. When exchangeable ions are H^+^, Brønsted acid sites are also present [8]. The strength of Lewis basic sites (12-ring oxygens in the windows of the supercages) in X and Y type zeolites are known to be dependent on the ion type [25]. For instance, the H^+^ selectivity was higher in X than in Y due to the variations in Si/Al ratios [12]. Schulthess *et al.* [15] reported the adsorption strength of Na^+^ was weak with zeolite Y but strong with mordenite zeolite. Differences in Si/Al ratios within and among zeolite species resulted in significant variations in their H^+^ adsorption selectivity.

Geometrical changes due to Si/Al ratio result in strong or weak electrostatic attractions depending on the distance of the framework charge sites and hence influence on the degree of H^+^ adsorption selectivity. It has been shown that the selectivity and maximum cation exchange level in zeolites with higher Si/Al ratio depend mainly on the separation distances of the framework charge sites [26]. The separation distance between framework charges is certainly an important parameter. Short separation distances means that two cations are located close to each other, which is unfavorable because of their electrostatic repulsion. Protons are not really bound like a cation, but are in covalent form [27] and do, therefore, not undergo such a strong electrostatic repulsion with neighbouring cations.

The overall charge strength and density is thus determined by the sum of the formal valences of the individual zeolite atoms *i.e.*, replacement of Si by Al results in a decrease in the charge of the cluster by one unit which is compensated by cations such as Na^+^ ions in the actual zeolites [28]. Thus changes in the Si/Al ratio result in variations in the magnitude of charge density and distribution that affect the selectivity of H^+^ or cation adsorption behavior.

The negative charge densities of the zeolite samples are also different from each other. High negative charge density of a zeolite makes negative charge sites be very close together resulting in increased or stronger electrostatic attraction forces and hence increase adsorption affinity [29]. While for a low negative charge density zeolite like mordenite, charge sites are far apart so the attractions are weak. As the negative charge density changes, the behavior or properties of the zeolites are significantly altered [22]. Furthermore, the surrounding atoms within the zeolite’s structural framework affects the electrostatic potential and the strength of binding interaction of cations [24].

Comparing the crystal structures of the five zeolites used in this study, they have different pores of different diameters within and among crystal structures (4A: 11.4 Å, 6.6 Å, 4.1 Å and 2.2 Å; X and Y: 13 Å, 8 Å, 7.4 Å, 7.3 Å, 4.8 Å and sodalite cages; Na-P1: 4.6–6.0 Å; mordenite: 7.0 Å, 4.2 Å and 3.6 Å) [3,4,30,31]. Differences in the pore sizes of various zeolites have an effect on the coexistence of extra-framework cations. Usually Na^+^ in aqueous solution is in hydrated form and may be weakly held or adsorbed onto zeolites depending on the pore size and location of charge site in the zeolite structural framework [32]. Sodium (1.9 Å) [33] and H^+^, which exists as a hydronium ion with effective ionic radius of 1.0 Å [34] in aqueous solution, have different ionic size and as such they have different affinities for the negative charge sites in zeolites. These exchanging ions are bound to the negative charge sites in zeolites by different mechanisms which make their exchange behavior different. Sodium is bound in zeolites through electrostatic attraction force [35] while H^+^ is usually bound through hydrogen bonding [36], and the hydrogen bonding becomes very strong as it binds to the negative charge site in the zeolite. Due to Lewis acid-base reactions, the formed hydrogen bonding through the bridging oxygen of the Al–O–Si linkage in the zeolite structural framework eventually results in a covalent bonding to form Al–OH–Si as has been described earlier. The formed covalent bonding is very strong as opposed to the electrostatic force between Na^+^ and negative charge. The hydrogen bonding is much stronger in high charge density zeolites like 4A than in low charge density zeolite like mordenite as such H^+^ easily replaced Na^+^. The nature of exchanging ions and the type of their binding energy to the negative charge sites makes zeolites more selective for H^+^ than Na^+^ and hence strong H^+^ selectivity by zeolites.

In addition to the above factors, the difference in the framework type and local fine structure of zeolites should be related to the different proton selectivity of Na-P1 type zeolite than the other zeolites (Figure 4), but it is difficult to explain the reason at this stage. Theoretical study such as quantum chemical calculations is needed to clarify the effect of the framework type and local fine structure on the proton selectivity of Al–O–Si group of zeolites

## 3. Experimental Section

The CEC measurements were conducted in the Laboratory of Applied Chemistry for Environmental Industry, Faculty of Agriculture, Ehime University, Matsuyama in Japan. Five different zeolite species with different Si/Al ratios, crystal structures and amount of charge densities were used in this study that include 4A, X, Y, Na-P1 and mordenite type zeolites. Chemical reagents and 4A, X, Y and mordenite type zeolite samples were purchased from Wako Chemicals Ltd, Osaka, Japan while Na-P1 zeolite samples with different Si/Al ratio were synthesized in this study.

### 3.1. Preparation of Na^+^-Saturated Zeolites

In a 250 mL centrifuge bottle, the respective zeolites (10 g) were washed five times with 1 M NaCl (150 mL) to saturate the zeolites with Na^+^. Then the content was washed twice with water (150 mL), and once with acetone (100 mL), air-dried, and used as a sample. The Na^+^ contained in the samples is exchangeable Na^+^ and free Na^+^ as NaCl. The sum of exchangeable and free Na^+^ contents of the sample (hereafter Na^+^ content) was determined by washing the sample (1 g) with 1 M NH_4_Cl (30 mL) seven times. Water content of the sample was determined by heating at 105 °C for 3 h. The content of Si and Al of the samples was determined after dissolution with hydrofluoric acid.

### 3.2. CEC Determination of Zeolites at Different pH

One gram of a sample was put into a 250 mL centrifuge bottle, then distilled water (50 g) was added. The contents were mixed well and left to stand for 1 h. Thereafter, distilled water and 10 mM HCl were added to give a total volume of 200 mL and HCl concentrations between 0 to 7.5 mM. The mixture was shaken for 3 h at 25 ± 0.5 °C, centrifuged at 2000 *g*, and the concentration of Na^+^ and pH of the supernatant were measured. Preliminary experiments indicated that the Na^+^ concentration and pH of the supernatant became constant within 3 h, and the measured Na^+^ concentration and pH are hereafter referred to as equilibrium Na^+^ concentration and equilibrium pH, respectively. CEC (the amount of Na^+^ retention) of a sample was simply calculated from the difference between Na^+^ content of the sample and the amount of Na^+^ in the supernatant. The concentration of Al and Si in the supernatant was measured to check the dissolution of the sample and the measurement of Na, Al and Si was carried out using atomic absorption spectrophotometer (Z-5000, Hitachi, Tokyo, Japan). Powder XRD pattern of the samples before and after the CEC determination was obtained with an Ultima IV X-ray diffractometer (Rigaku, Tokyo, Japan) with Cu-Kα radiation generated at 40 kV and 40 mA, between 3–60° of 2θ angles with a sampling width of 0.02° and a scanning rate of 2° min^−1^.

## 4. Conclusions 

The amounts of Na^+^ adsorption on 4A, X, Y, Na-P1 and mordenite type zeolites in aqueous media, in a two-cation (Na^+^ and H^+^) system, were largely dependent of the pH−pNa value of the aqueous solutions, and the dependence was different between the zeolites. Zeolites with lower Si/Al ratios and therefore with higher negative charge density (4A and X type zeolites), had greater selectivity for H^+^. The H^+^ selectivity had a good relation with the Si/Al ratio of zeolites, regardless of the difference in the framework type of the zeolites, indicating the Si/Al ratio is the main cause of the H^+^ selectivity of zeolites. Because such low Si/Al ratio zeolites with greater CEC begin to adsorb H^+^ even at alkaline pH values, the use of low Si/Al ratio zeolites for the purpose of water decontamination needs to be performed with care, because the adsorption of H^+^ is usually followed by the dissolution of zeolites.
